# Supplementary material for: Patterns of Intron Gain and Loss in Fungi
Source: PLoS Biol. 2004 Nov 30;2(12):e422. doi: 10.1371/journal.pbio.0020422 (PMC532390; doi:10.1371/journal.pbio.0020422)
Supplement: Table S1 — Also available at http://genes.mit.edu/NielsenEtAl/. (4.3 MB ZIP). [file pbio.0020422.st001.zip › NielsenEtAl/html/59.html]

AN0406.1.NCU00311.1.MG06334.1.FG05377.1


```
 CLUSTAL W (1.82) Multiple Sequence Alignments - Introns Inserted


Sequence 1: NCU00311.1	610 aa
Sequence 2: MG06334.1	613 aa
Sequence 3: FG05377.1	617 aa
Sequence 4: AN0406.1	611 aa
Alignment Length: 667 aa
Number Identitical Residues: 322 aa
Alignment Score (without introns) 13810


MG06334.1 	MSAAQSTATNRMSTPEASNTHAASNRMSTPEASNVSSLRPPSSRAVGSGSHLRASADVAA
NCU00311.1	-----------MSN-------IMSNRNSTPEASNVSSLRPPSSRAIGSNHGLRASADVAA
FG05377.1 	-----------MSG-----NHVIGNRNSTPEANNTSTLRPPSSRAIGSGHSLRASADMAS
AN0406.1  	-----------MAS------HIIGNRNSTPEAS-KSSLRPPSSSRNLATHQLRASADMSG
          	           *:          .** *****.  *:******    :   ******::.

MG06334.1 	LTGSAARGGNARPSSDFYHG------QGNSEIDPQDKLAQQWIADIDQYETTLEEMAAAT
NCU00311.1	LTGAQAAASRIRPSSDFYGQPQSGQVQGNAELDPQDKLAQQWIADIDQYETTLEEMAAAT
FG05377.1 	LTGPSP-SNRIRPSSDFYGQAQQNLGPNNAESDSQDKIAQQWIADIDQYETTLEEMAAAT
AN0406.1  	FPSPLS-SRSIRPASEVYFN-QQAQTPGNAE-DPLDRAAQQWLADIDQYETTLEEMAAAT
          	:... . .   **:*:.*   ..    .*:* *. *: ****:*****************

MG06334.1 	LDQDFKDELSAIEQWFRVLSEAERTAALYALLQQTTQVQIRFFIQVLQQMGKNHPMSGVL
NCU00311.1	LDQDFKDELSAIEQWFRVLSEAERTAALYALLQQTTQVQIRFFIQVLQQMGKNHPMSGVL
FG05377.1 	LDQDFKDELSAIEQWFRVLSEAERTAALYALLQQTTQVQIRFFIQVLQQMGKNHPLSGVL
AN0406.1  	LDQDFKDELSAIEQWFRVLSEAERTAALYALLQQTTQVQIRFFIQVLQQMAKSHPMSGLL
          	**************************************************.*.**:**:*

MG06334.1 	SPANFDK--~-D1PMSNRLSDAMNKLSVDSARNSIARG-PASQGAKRQSGLDPSTINMMF
NCU00311.1	SPANFDK--~-D1PMSSRLSDAMGKLEVGSARNSLAR--PSGGANKRHSGLDQTTINTMF
FG05377.1 	SPASFDK--~-D1PMSNRLSDAMNKLNVDSARNSMARSSVIAPSTKRHSGLDPTTISAMF
AN0406.1  	SPANFGEKG1ID~AMSNRLNDAMSKLNVDSSRNSLGRP-PPSPGAKRNSGLDSSTINAMF
          	***.*.:..  * .**.**.***.**.*.*:***:.*    . . **:**** :**. **

MG06334.1 	PDAAAAIATEKAKFTQQTGNQPPSNRNSTAVDNRNSLVGASEG---NDANG--QHPTSPW
NCU00311.1	PDAVAAIATEKAKFTQQTGNPPPSNRNS-LVDNRNSLAAPTVSGPKDDING--QNPASPW
FG05377.1 	PDAAAAIATEKAKFTQQTGNPPSSNRNSIALDHRSSMAAPSISAPQDGRDAG-PAGSSPW
AN0406.1  	PDAAAAIAKKKAEFTQQTGNAPPSNRNSAVFNERTSFVAPTISAPDNSADNLSQPPVSPW
          	***.****.:**:******* *.*****  .:.*.*:...: ....:. :  :    ***

MG06334.1 	GTG-DAAA-----RAKPAPGQAPMGQFAAPSIGAG-LRSPRPGQLGGNAQIQNTTLTASD
NCU00311.1	GPS-DTS------RPKSSTGQTPMGQFVQPPPSAGGLRSPRPPQLSSNTNIQSTTLTAAE
FG05377.1 	NSGGNGDT-----SAKPSSSQTPMGQFVQPTPSGG-LRSPRP-QLSSNNTIQQTTLNAPD
AN0406.1  	AQRGASEPQPPIARPKSSSGQQPMGQFNQ----SG-LRSPLP-------TSQTATIPAP-
          	   .   .... : .*.:..* *****      .* **** *         * :*: *. 

MG06334.1 	KPGGELGVASPFGGPSGGNWASMVNTPMAANFPQQQ----GGQADMLANATAMKLAAMST
NCU00311.1	PQLTELPLLSPY--TSSGNWASMVNTPMVPTFGQTQ----GNNADMVANATAMKLAALST
FG05377.1 	KTPADLPLLSPYN-AGSGNWASMVNTPMTSTFNAAATG--GNQADMVANATAMKLAALST
AN0406.1  	--EIEAPLLSPYN--VNASWASMTNTPMTATFGSQLGAPHQQGSDMVANATAMKLAALST
          	    :  : **:.   ...****.****...*     ..    :**:**********:**

MG06334.1 	VNNRFALDDVRKYRRTRSNDNS-----GQNPLSPG------MISPNVVMVDEHGRVLSRD
NCU00311.1	VNNRFALDDARRYRRARSNDGAPGQSHAQHPLSPGPQ---GIPSTNVVMINEHGQVLSHD
FG05377.1 	VNNRFALDDVRKYRRARSNDGTP----GQNPVPTGPQPGINMPNANVVMINEHGQVLSRE
AN0406.1  	VNNRIALDDARKYRRARSNDGQG----KNASNNTGAQS--IQGGLASPGLPVAGQLLNAQ
          	****:****.*:***:****.       : .  .*...     .     :   *::*. :

MG06334.1 	QLLNMQGMQSQ--QQ--MSFN-NRSRPNSPGIALQQAAGFGGMAFASPQSNNFMLG---N
NCU00311.1	HVLALQQQQQQGLQQGLGGFGGHRSRPTSPGIAMPNYG--SALQFTSPQNNGFLSAYDGG
FG05377.1 	QIMALQAQQSL-------GMGGQRSRPSSPGLAMQPGLA-HMAPFTSPQNNGFLSAYDVS
AN0406.1  	QFAALQAQQQA-------AMAGHRSRPTSPGIAMQGGAL-GPMGFTSPQNNGFLTAYDPN
          	:.  :*  *.        .: .:****.***:*:          *:***.*.*: . . .

MG06334.1 	ALNPMSGGLAPVGMGQFGMGGLAPEGYLSDHSDMNRGRSPRGRRGSSKPPEDPTDPTLLQ
NCU00311.1	APGLINNGLVPM-MGQFNLGGHHAEGYLSDHSEINRGRSPRGRRGSSKPPEDPTDPTLLQ
FG05377.1 	SP-LLSAGMQSVNMGSLGMGGH--EGYLSDHSDMVRGRSPRGRRGSSKPPEDPTDPTLLQ
AN0406.1  	NP-LIGNGLGALGMGQFGLSGH--EGYLSDHSEINRGRSPRGRRGSSKPPEDPTDPNLLK
          	    :. *: .:.**.:.:.*   ********:: *********************.**:

MG06334.1 	DIPAWLRSLRLHKYTDNLKDMRWVDLIELDDKQLEERGVNALGARRKMLK~VFEQVKV1Y
NCU00311.1	DIPSWLRSLRLHKYTDNLKDMKWTDLIELDDKQLEERGVNALGARRKMLK~VFEQVKG1-
FG05377.1 	DIPSWLRSLRLHKYTDNLKDMKWTDLIELDDKALEERGVNALGARRKMLK~VFEQVKE1A
AN0406.1  	DIPSWLRSLRLHKYTDNLKDLKWTELIELNDKQLEERGVNALGARNKMLK0VFEQVKE~A
          	***:****************::*.:****:** ************.**** ******   

MG06334.1 	AR---------
NCU00311.1	-----------
FG05377.1 	KADGKLG----
AN0406.1  	KAEGKLDNATA
          	  ... ..:::
```
